# Multivariate synthesis optimization, comprehensive characterization, and surface ligand determination of palladium nanoparticles

**DOI:** 10.1038/s41598-026-69103-3

**Published:** 2026-09-02

**Authors:** Eduardo Sidinei Chaves, Morgana Lurdes da Rocha, Isabella Tavernaro, Ute Resch-Genger, Björn Meermann

**Affiliations:** 1 Department of Chemistry, Federal University of Santa Catarina, Florianópolis, Brazil; 2 Federal Institute for Materials Research and Testing, Division 1.2- Biophotonics, Berlin, Germany; 3 Federal Institute for Materials Research and Testing, Division 1.1- Inorganic Trace Analysis (ITALab), Berlin, Germany

**Keywords:** Metallic nanoparticles, Broad particle size distribution, Real-world nanomaterials, Ligand density, ICP-MS, Complementary NPs characterization, Chemistry, Nanoscience and technology

## Abstract

We present a high-throughput approach for synthesizing palladium nanoparticles (PdNPs), which are widely used as catalysts in industrial processes, employing an aqueous reaction medium and a commercial reaction platform that enables parallel reactions under identical conditions. The optimal synthesis conditions, including reaction temperature and the concentrations of Pd, thiol ligands (3-mercaptopropionic acid (MPA) and L-cysteine (Cys)), and reducing agent, were established using a Doehlert experimental design. The purified thiol-capped PdNPs were characterized by transmission electron microscopy (TEM), energy-dispersive X-ray spectroscopy (EDS), dynamic light scattering (DLS), and nanoparticle tracking analysis (NTA), confirming the formation of irregularly shaped PdNP-MPA and PdNP-Cys with polydispersity indices up to 0.270. Single particle-inductively coupled plasma-mass spectrometry (sp-ICP-MS) enabled determination of particle size and size distribution, demonstrating its suitability for characterizing polydisperse nanoparticles with irregular shapes and yielding results consistent with those obtained by TEM and NTA. Bulk ICP-MS was employed to determine the surface density of thiol ligands from the sulfur-to-palladium ratio. Overall, this study demonstrates the potential of multivariate experimental design for PdNP synthesis and the value of complementary analytical techniques for comprehensive nanoparticle characterization.

**Supplementary Information:**

The online version contains supplementary material available at https://doi.org/10.1038/s41598-026-69103-3.

## Introduction

Engineered nanomaterials (NMs) with tailor-made architectures and surface chemistries enabled by ligand design have been increasingly used in medical diagnostics^1,2^, energy storage and conversion^3^, optoelectronics^4^, catalysis^5^, environmental pollutant removal^6^ and chemical modifiers^7,8^. Examples include metal nanoparticles (NPs), such as silver, gold^9^, and palladium^10^, which can be produced in different sizes, shapes, and surface chemistries^11^. However, palladium-based nanoparticles (PdNPs) stand out and have been successfully applied in environmental analysis using graphite furnace atomic and molecular absorption spectrometry, respectively, for the determination of Pb in petroleum waste leachate^7^ and for sulfur fractionation in coal samples^8^. PdNPs also have demonstrated high efficiency as catalysts for Suzuki-Miyaura cross-coupling reactions, selective alcohols oxidation and reduction of Cr(VI) to Cr(III), as well as revealing excellent antimicrobial and anti-biofilm activities^12^. Although the synthesis of NPs, particularly PdNPs, can be performed by well-established physical or chemical methods^10^, the need for more sustainable and environmentally friendly methods using low-toxicity reagents and stabilizing agents remains^12,13^.

Synthesizing tailored NPs by optimizing several parameters using a conventional univariate approach is typically time-consuming and requires a high number of experiments^14,15^. These approaches are, in general, not sustainable, requiring high energy and chemical consumption and contributing to waste generation. This renders multivariate optimization very attractive and environmentally friendly, enabling the effective optimization of tailored NPs with a reduced number of experiments and maximizing the information obtained^14,16,17^. The advantages of the multivariate optimization approach can be enhanced by combining it with synthesis devices enabling the performance of many reactions with differing reaction mixtures in parallel. A fast screening of the main significant parameters with a reduced number of experiments is feasible with the fractionated factorial design approach, while, e.g., a surface response methodology such as the Doelhert design, allows for the determination of the optimal experimental conditions to obtain the NPs^15,18^.

Next to synthesis, the wide range of applications of tailored NPs^1–8^, highlights the importance of NPs characterization, particularly in terms of size, shape, particle number concentration (PNC), composition and chemical surface for understanding their uptake, fate, transport, and toxicological effects, as well as for increasing NMs safe-by-design concepts^19^. Therefore, complementary analytical methods for a holistic characterization of NPs are fundamental for quality control assessment as well as avoiding harmful effects of these NMs^20^.

A variety of analytical techniques have been employed to characterize NMs in terms of size, shape, PNC, composition, and surface properties^21^. Among these, dynamic light scattering (DLS), transmission electron microscopy (TEM), nanoparticle tracking analysis (NTA), asymmetrical flow field flow fractionation (AF4), small-angle X-ray scattering (SAXS), single particle-inductively coupled plasma-mass spectrometry (sp-ICP-MS), particle-induced X-ray emission (PIXE)^21–24^ are well established. In addition, surface-related properties and particle ligand interactions can be accessed by surface plasmon resonance (SPR)^25^, fourier-transform infrared spectroscopy (FTIR)^26^, and magnetic resonance spectroscopy (NMR) analysis^27^. For metallic NPs functionalized with compounds containing heteroatoms, such as S and P, the ligand density has been successfully determined by bulk ICP-MS analyses, through monitoring both the metallic core and the heteroatom associated with the surface ligands^28^. Additionally, optical assays, potentiometric, and thermogravimetric methods have been employed for quantifying the surface groups in different NPs^27,29,30^. However, since many analytical methods for NPs characterization are based on models that assume spherical and/or monodisperse particles, characterizing polydisperse and irregularly shaped NPs remains a challenge.

In this sense, we present a multi-method approach for the complementary characterization of metallic NPs. Considering the widespread use of PdNPs in catalysis and industrial processes, as well as their environmental relevance^7,8,10,12^, PdNPs capped with MPA and Cys were synthesized using a commercial multi-synthesis approach and employed as proof-of-concept particles for the complementary characterization of NPs. The proposed synthesis was carried out in aqueous medium using a multivariate optimization approach to establish the compromise synthesis conditions.

## Results and discussion

**Fig. 1:**
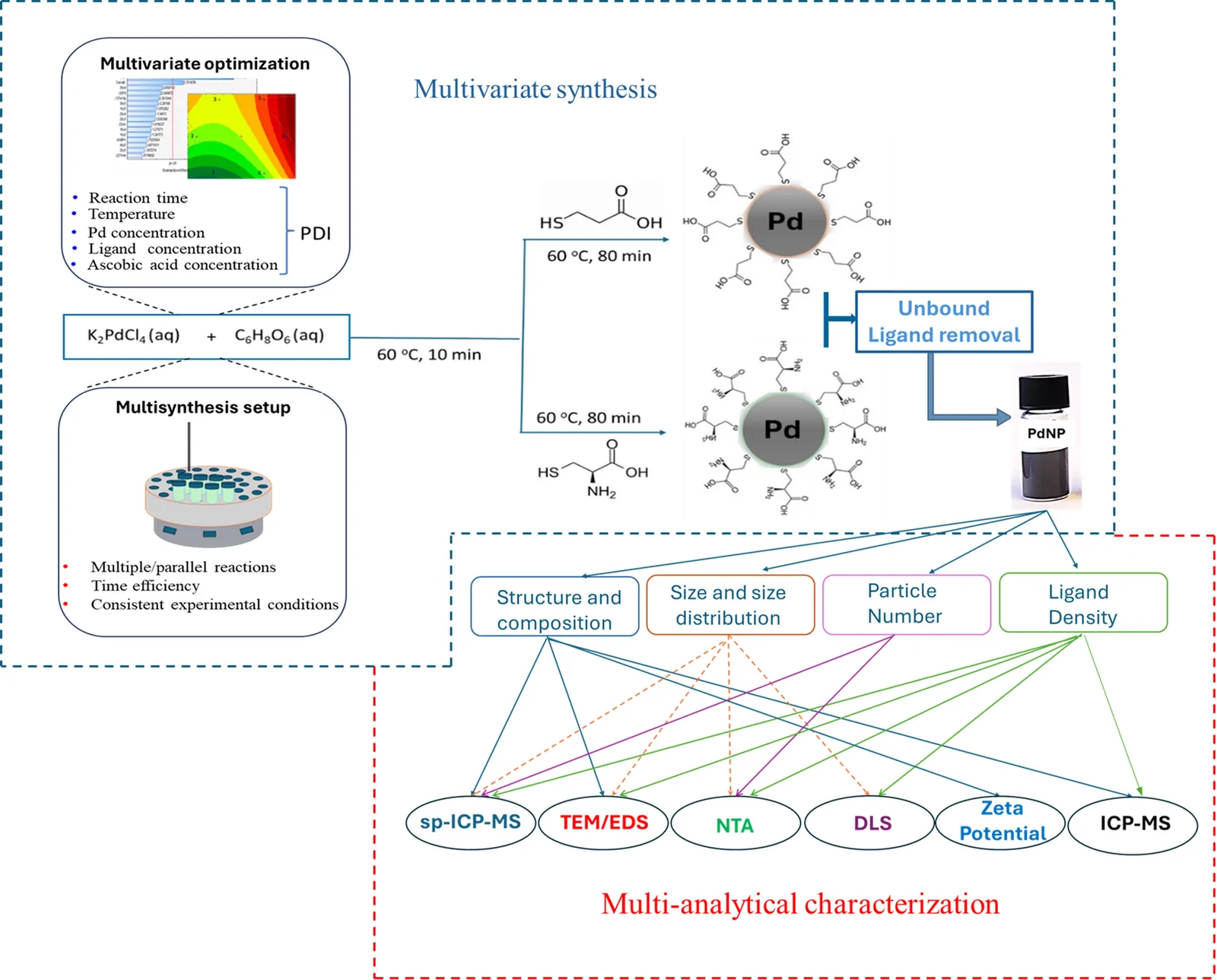
Workflow of the synthesis of PdNP-MPA, and PdNP-Cys, purification for unbounded ligand removal, multi-analytical approach for characterization and ligand density assessment.

The proposed synthesis of the PdNPs represents an environmentally friendly alternative to conventional methods, particularly when combined with a commercial multi-synthesis device for running multiple reactions in parallel and a multivariate optimization, reducing reagent consumption and experimental waste. The optimization of these parameters is highly important to promote an analytically accessible and standardized PdNPs synthesis procedure, minimizing the uncontrolled broadening of the size distribution. A brief overview of the synthesis and characterization of the thiol-capped PdNPs is demonstrated in the workflow in Fig. 1.

The synthesized PdNPs were thoroughly purified to remove unbound ligands, prior to characterizing their size, size distribution, shape, PNC, and composition, through a complementary analytical technique. The total Pd and S concentrations were determined by ICP-MS. The concentrations determined were applied for the thiol ligand density determination on PdNPs surface.

### Nanoparticles synthesis using multivariate optimization

Multivariate optimization for NPs synthesis was performed first by fractionated factorial design 2^5−1^, for screening of the significant parameters for NPs synthesis (*p* < 0.05) as well as the potential interplay of parameters in the evaluated range, followed by response surface methodology (Doehlert design). The applied 2⁵⁻¹ design matrix and its corresponding responses (1–PDI) are presented in Table 1. As observed, for the evaluation experimental uncertainty and reproducibility of experiments the central point carried out in triplicate, minimizing the total number of experiments and preserving the statistical robustness of the model.

**Table 1:** Experiments, real and coded values (in parentheses) for the 2⁵⁻¹ fractional factorial design for screening the synthesis parameters of PdNPs using 3-mercaptopropionic acid (MPA) as ligand.

Experiment	Temperature [^o^C]	Time [min.]	Pd [µmol]	Ligand [mmol]	Ascorbic Acid [mmol]	1-PDI*
1	25 (-1)	30 (-1)	2 (-1)	0.02 (-1)	0.25 (+ 1)	0.800
2	25 (-1)	30 (-1)	2 (-1)	0.14 (+ 1)	0.05 (-1)	0.793
3	25 (-1)	30 (-1)	16 (+ 1)	0.02 (-1)	0.05 (-1)	0.700
4	25 (-1)	30 (-1)	16 (+ 1)	0.14(+ 1)	0.25 (+ 1)	0.731
5	25 (-1)	180 (+ 1)	2 (-1)	0.02 (-1)	0.05 (-1)	0.802
6	25 (-1)	180 (+ 1)	2 (-1)	0.14 (+ 1)	0.25 (+ 1)	0.792
7	25 (-1)	180 (+ 1)	16 (+ 1)	0.02 (-1)	0.25 (+ 1)	0.711
8	25 (-1)	180 (+ 1)	16 (+ 1)	0.14 (+ 1)	0.05 (-1)	0.755
9	75 (+ 1)	30 (-1)	2 (-1)	0.02 (-1)	0.05 (-1)	0.719
10	75 (+ 1)	30 (-1)	2 (-1)	0.14 (+ 1)	0.25 (+ 1)	0.752
11	75 (+ 1)	30 (-1)	16 (+ 1)	0.02 (-1)	0.25 (+ 1)	0.818
12	75(+ 1)	30 (-1)	16 (+ 1)	0.14 (+ 1)	0.05 (-1)	0.838
13	75 (+ 1)	180 (+ 1)	2 (-1)	0.02 (-1)	0.25 (+ 1)	0.747
14	75 (+ 1)	180 (+ 1)	2 (-1)	0.14 (+ 1)	0.05 (-1)	0.631
15	75(+ 1)	180 (+ 1)	16 (+ 1)	0.02 (-1)	0.05 (-1)	0.833
16	75 (+ 1)	180 (+ 1)	16 (+ 1)	0.14 (+ 1)	0.25 (+ 1)	0.841
17	50 (0)	105 (0)	9 (0)	0.080 (0)	0.15 (0)	0.833
18	50 (0)	105 (0)	9 (0)	0.080 (0)	0.15 (0)	0.824
19	50 (0)	105 (0)	9 (0)	0.080 (0)	0.15 (0)	0.805

(-1) minimum coded values; (+ 1) maximum coded values; (0) central point values.

*PDI: polydispersity index; 1-PDI transformation adopted for convenience to facilitate the interpretation of the response.

Considering a confidence level of 95%, the effect of the selected parameters and their interactions was assessed by analysis of variance (ANOVA) and the Pareto´s chart (Figure S1). The ANOVA table, showing significant effects and the determination coefficient (R²) for the evaluated parameters of the 2^5−1^ factorial design, is presented in Table S1. The reaction time, temperature and the concentrations of Pd, ligand and reductant agent as well as most of their interaction did not significantly affect the size, size distribution, and shape of the obtained PdNPs. Furthermore, the curvature and the interplay between temperature and Pd concentration showed a positive and significant (*p* < 0.05) effect over the PDI of the synthesized PdNPs, suggesting that the optimal synthesis condition may be included in the evaluated range. Thus, since the interaction between temperature and Pd concentration showed a significant effect, a response surface methodology (Doehlert design) was applied to determine the optimal conditions for the PdNPs synthesis. The applied Doehler design matrix and the corresponding responses based in 1–PDI are presented in Table 2.

**Table 2:** Experiments, real and coded values (in parentheses) for Doehlert design of the PdNPs synthesis capped with 3-mercaptopropionic acid (MPA) and L-cysteine (Cys).

Experiment	Factors		MPA		Cys	
	X1(Pd, [µ mol])	X2 (T, [^o^C])	PDI	1-PDI	PDI	1-PDI*
1	10 (-1)	60 (0)	0.237	0.763	0.181	0.819
2	15 (-0.5)	40 (-0.866)	0.244	0.756	0.229	0.771
3	15 (-0.5)	80 (0.866)	0.201	0.799	0.212	0.788
4	25 (-0.5)	40 (-0.866)	0.239	0.761	0.258	0.742
5	25 (-0.5)	80 (0.866)	0.205	0.795	0.297	0.703
6	30 (1)	60 (0)	0.165	0.835	0.347	0.653
7	20 (0)	60 (0)	0.243	0.757	0.289	0.711
8	20 (0)	60 (0)	0.222	0.778	0.293	0.707
9	20 (0)	60 (0)	0.192	0.808	0.355	0.645

*PDI: polydispersity index; 1-PDI transformation adopted for convenience to facilitate the interpretation of the response.

**Fig. 2:**
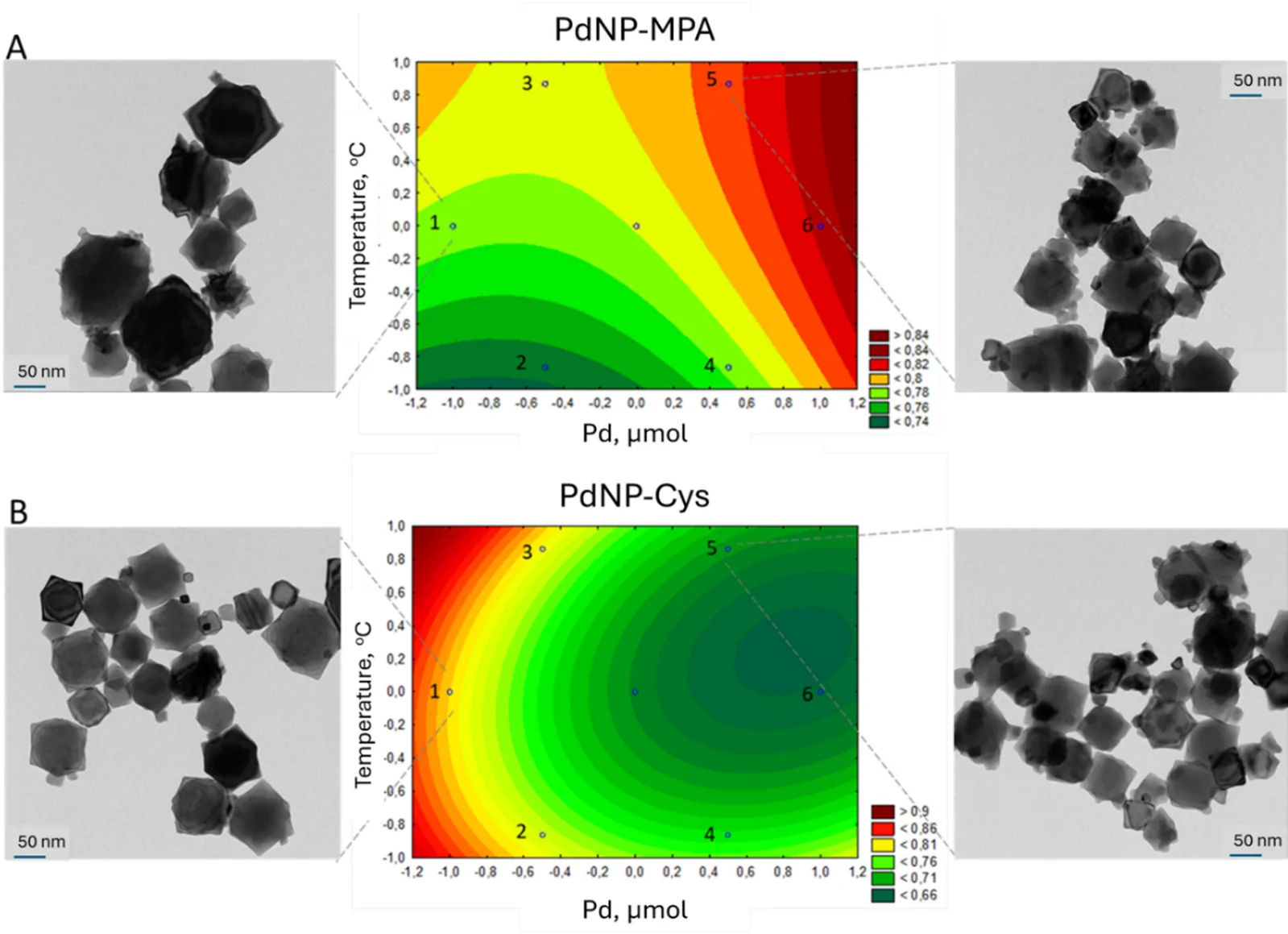
Contour surfaces and TEM images of nanoparticles obtained under the conditions of experiments 1 and 5 for (**A**) PdNP-MPA and (**B**) PdNP-Cys synthesis using Doehlert design optimization. The numbers on the surface correspond to the experimental runs of the Doehlert design. The color scale ranges from green (higher PDI) to red (lower PDI).

For the Doehlert design optimization, the effect of temperature and Pd concentration over the PDI of the synthetized PdNP-MPA and PdNP-Cys were evaluated. The obtained contour surface for (A) PdNP-MPA and (B) PdNP-Cys using a Doehlert design optimization and the respective TEM images for the selected synthesis conditions of experiments 1 and 5 are shown in Fig. 2.

The ANOVA for both PdNPs (Tables S2 and S3) shows no significant effect of the evaluated parameters in the range considered. These results imply that any of the evaluated conditions employed in the Doehlert design experiments could be applied to PdNPs synthesis. However, comparing TEM images of PdNPs synthetized at moderate temperature and a low Pd concentration (60 °C, 10 µmol ‒ experiment 1) with those obtained at high temperature and Pd concentration (80 °C, 25 µmol ‒ experiment 5), under the latter conditions there is a higher aggregation, with small nanoparticles attached to the surface of larger ones. On the other hand, under the conditions used in experiment 1, the concentration of smaller particles is slightly minimized leading to more uniform particles. Accordingly, since optimal conditions could not be clearly defined, the following compromise experimental conditions were selected for the synthesis of PdNPs: 10 µmol of Pd, 0.15 mmol of ascorbic acid as reducing agent, 0.08 mmol of ligand (MPA or Cys), temperature of reaction of 60 ± 3 °C for 90 min. Afterwards, PdNPs obtained under the compromise conditions underwent a purification step to remove the unbound ligands, performed through washing cycles, monitoring the concentration of S in the ultrafiltrate solutions (Figure S2). From the 6th to the 8th washing cycle the concentration of S in the washing supernatant did not change significantly (Anova, 95% of confidence level). Thus, six washing cycles were performed to remove the unbound ligands. This is in good agreement with the findings of Elzey et al. for unbound ligand removal from thiol-capped gold NPs^31^.

Overall, multivariate optimization combined with the application of a multi-synthesis setup enabled to efficiently define the compromise conditions for the PdNPs synthesis and to assess the parameters’ interplay with a reduced number of experiments. Under these synthesis conditions, the uncontrolled broadening of the NP size distribution is minimized, leading to a standardized procedure for PdNP synthesis. Additionally, compared to conventional procedures using univariate optimization and single batch reaction systems, the multivariate optimization and multi-synthesis approach markedly reduce the number of experiments required to establish the synthesis conditions. Consequently, this approach decreases the consumption of reagents, energy, and the generation of waste, characteristics that are aligned with the principles of green chemistry.

### Structure and composition

**Fig. 3:**
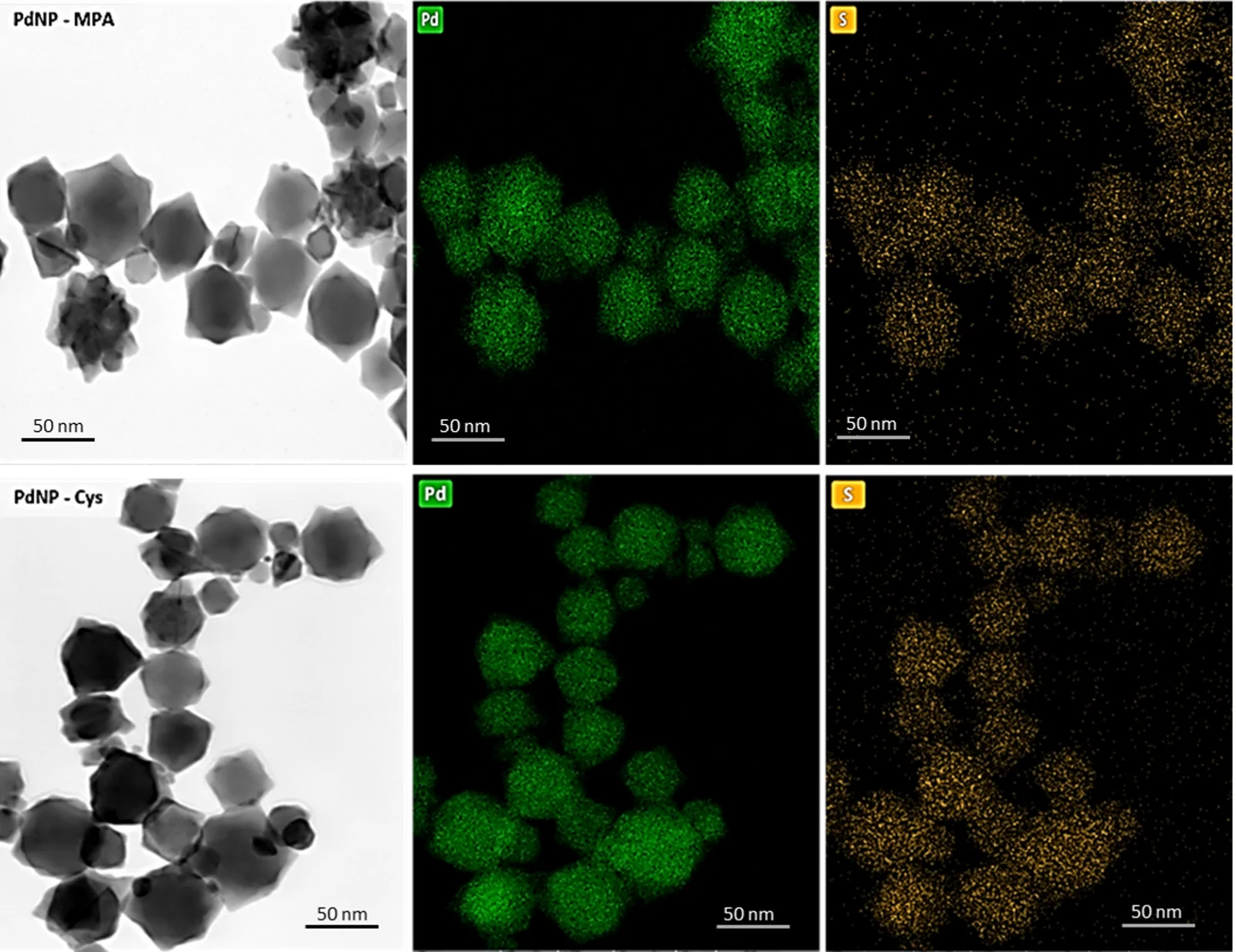
TEM images and EDS mapping of Pd and S for PdNPs capped with MPA and Cys.

The morphological and composition characterization of the synthetized PdNPs capped with MPA and Cys was performed by TEM/EDS analysis. The obtained TEM images and elemental mapping for Pd and S in the synthetized PdNPs are shown in Fig. 3.

As observed in the TEM images, both PdNP-MPA and PdNP-Cys samples contain NPs varying in size and shape. The measured mean size for PdNP-MPA and PdNP-Cys were 96 ± 33 nm and 71 ± 30 nm, respectively. The shape of both synthetized PdNPs is challenging to define since mixed shapes (e.g., cuboctahedron, truncated octahedrons, quasi-spherical, etc.), and some multi-pod particles were observed. Considering the proposed water-based synthesis and L-ascorbic acid as a reducing agent, the fast reduction of Pd ions may promote the formation of thermodynamically favorable species such as truncated octahedrons and multiple-twinned particles^32^. Therefore, under the applied synthesis conditions, the TEM images suggest a non-uniform nanoparticle growth.

Mapping of Pd and S reveals the distribution of these elements in PdNP-MPA and PdNP-Cys particles. No significant amount of S and/or Pd was detected in areas outside of the nanoparticles, indicating that no relevant amount of Pd ions or unbound ligand is present. As expected, the elemental mapping for both types of synthesized PdNPs shows that Pd is evenly distributed in each particle. The distribution of S for both PdNP-MPA and PdNP-Cys, which perfectly overlaps with the nanoparticle regions, suggests effective and uniform surface coverage by the thiol-based ligands. Using EDS analysis, the concentration of S on the PdNPs surface was also estimated. The analysis was performed over two to three nanoparticle regions, and the found S concentration in both PdNPs was comparable: PdNP-MPA (0.21 ± 0.11% (*w/w*)) and PdNP-Cys (0.14 ± 0.11% (*w/w*)). Although these results may have a high uncertainty, they confirm the presence of S on the particles´ surface. In addition, these results are in good agreement with those obtained after bulk ICP-MS analysis, where the total S concentration onto particles´ surface was determined to evaluate ligand density^31^, these results will be discussed in the section *Ligand density assessment.*

### Size, size distribution and suspension stability

The surface charge of the PdNP-MPA and PdNP-Cys which correlates with colloidal stability^21^ was evaluated by zeta potential analysis. The zeta potential of the analyzed samples ranged from -29.6 mV to -35.0 mV indicating the good stability of the PdNPs suspension. Next, hydrodynamic sizes and polydispersity index (PDI) for PdNP-MPA and PdNP-Cys were determined by DLS and NTA. The results are summarized in Table 3.

**Table 3:** Hydrodynamic size and surface charge properties for PdNP-MPA and PdNP-Cys.

	*Technique*	*Parameter*	*Mean size* *(nm)*	*PDI*
*PdNP-MPA*	*DLS*	*Intensity-based size*	*161 ± 2*	*0.270 ± 0.017*
	*NTA*	*Number-based size*	*143 ± 7*	*-*
*PdNP-Cys*	*DLS*	*Intensity-based size*	*111 ± 1*	*0.133 ± 0.018*
	*NTA*	*Number-based size*	*112 ± 4*	*-*

The DLS results show that PdNP-MPA has a slightly higher hydrodynamic diameter and PDI values compared to PdNP-Cys indicating that PdNP-MPA is more polydisperse. Though, according to a paired t-test at 95% confidence, there is no significant difference (p-value > 0.05) between the mean size obtained from DLS and NTA for PdNP-MPA and PdNP-Cys (Table S4). Moreover, compared to the number-based size distribution given by DLS (Figure S3), the NTA analyses allow for a better evaluation of the size distribution of the PdNP-MPA and PdNP-Cys as demonstrated in Figure S4. The mean size determined by NTA was comparable (PdNP-Cys) or slightly lower than those obtained using DLS, as observed for PdNP-MPA. Additionally, the NTA results confirm the presence of multiple sizes of NPs in PdNP-MPA and a more uniform distribution for PdNP-Cys. Overall, the PdNP-Cys show a narrower size distribution and a lower PDI, while in PdNP-MPA the particles comprise higher polydispersity.

**Fig. 4:**
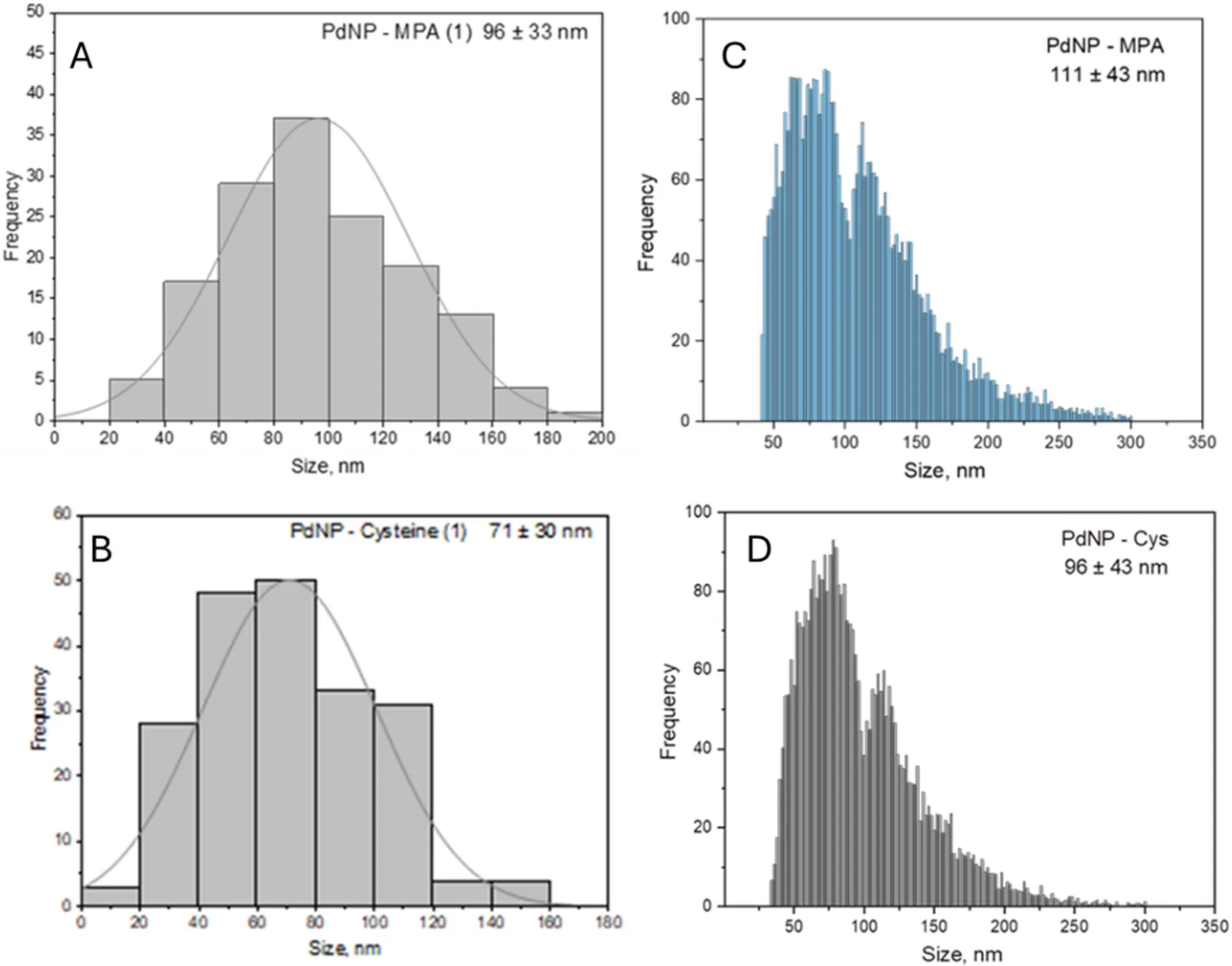
Mean size and size frequency distribution histograms obtained *via* TEM (**A** – PdNP MPA, **B** – PdNP Cys), and sp-ICP-MS (**C** – PdNP-MPA, **D** – PdNP-Cys).

The mean size and size distribution for both PdNP-MPA and PdNP-Cys was also assessed by high-resolution images of TEM analysis and element-specific determination using sp-ICP-MS. The mean sizes and obtained histograms are shown in Fig. 4.

The wide size distribution revealed by TEM and sp-ICP-MS were, in general, comparable and consistent with the results obtained by DLS, and NTA. However, comparing the mean sizes obtained by TEM for PdNP-MPA (96 ± 33 nm) and PdNP-Cys (71 ± 30 nm), the sizes were slightly lower than those obtained by sp-ICP-MS (PdNP-MPA 111 ± 43 nm and PdNP-Cys 96 ± 43 nm). Nevertheless, considering the limitations of 2D images for determining NPs size, some smaller or no uniform particles may be excessively represented in TEM analysis^33,34^. Furthermore, sp-ICP-MS takes thousands of PdNPs into account, while TEM is limited to a few hundred particles.

The size distribution obtained by sp-ICP-MS shows that complete size distribution for both PdNP-MPA and PdNP-Cys were not acquired, and a bimodal size distribution was obtained. This fact, combined with the broad size distribution of the synthesized NPs, suggests that particles smaller than 30 nm (sp-ICP-MS LOD_size_) might not have been considered in the calculation of the mean size and total particle number. Besides the size-dependent LODs, this fact may also be related to the restricted linear range, and physical issues during sample introduction *via* nebulization. Moreover, larger or aggregated particles may not be sufficiently ionized in the plasma, leading to a reduced sensitivity (and hence, wrong size allocation), and recovery rates^35^. Consequently, these facts can affect the determination of the total particle number and the average size as discussed by Gimenez-Ingalaturre et al. (2023)^36^.

### Particle number concentration by sp-ICP-MS

To assess the accuracy sp-ICP-MS measurements for PNC and for size determination, the analysis of a platinum nanoparticles (PtNPs) reference material was performed (Figure S5-A). The results obtained by sp-ICP-MS for size (49 ± 1 nm) and PNC (3.5 × 10^13^ ± 2.5 × 10^12^ particles/L) showed a good agreement with those found in the PtNP certificate (51 nm and 4.0 × 10^13^ particles/L). The quality of the size distribution and particle counting performed by sp-ICP-MS depends on the proper sample dilution. According to Gimenez-Ingalaturre et al. 2023, performing the analysis with different dilutions enables the selection of appropriate sample dilution to ensure quality control for size, size distribution and PNC^36^. Thus, the accuracy PdNP-MPA and PdNP-Cys analysis performed by sp-ICP-MS was assessed under different dilutions (Figure S6). As can be seen, considering the evaluated dilutions (100 × 10^3^ to 1000 × 10^3^ times), the particle size determined for both PdNP-MPA and PdNP-Cys did not change expressively. However, it was noticed that the results for particle counting were affected for both PdNPs when dilutions higher than 200 × 10^3^ times were applied. Taking this into account and keeping acquisition time (180 s) constant, the observed variation in PNC may be associated with statistical issues due to the low number of detected particles^36^. Thus, a dilution up to 200 × 10^3^ times is applicable for the determination of size, size distribution and particle number concentration of the proposed PdNP-MPA and PdNP-Cys.

**Fig. 5:**
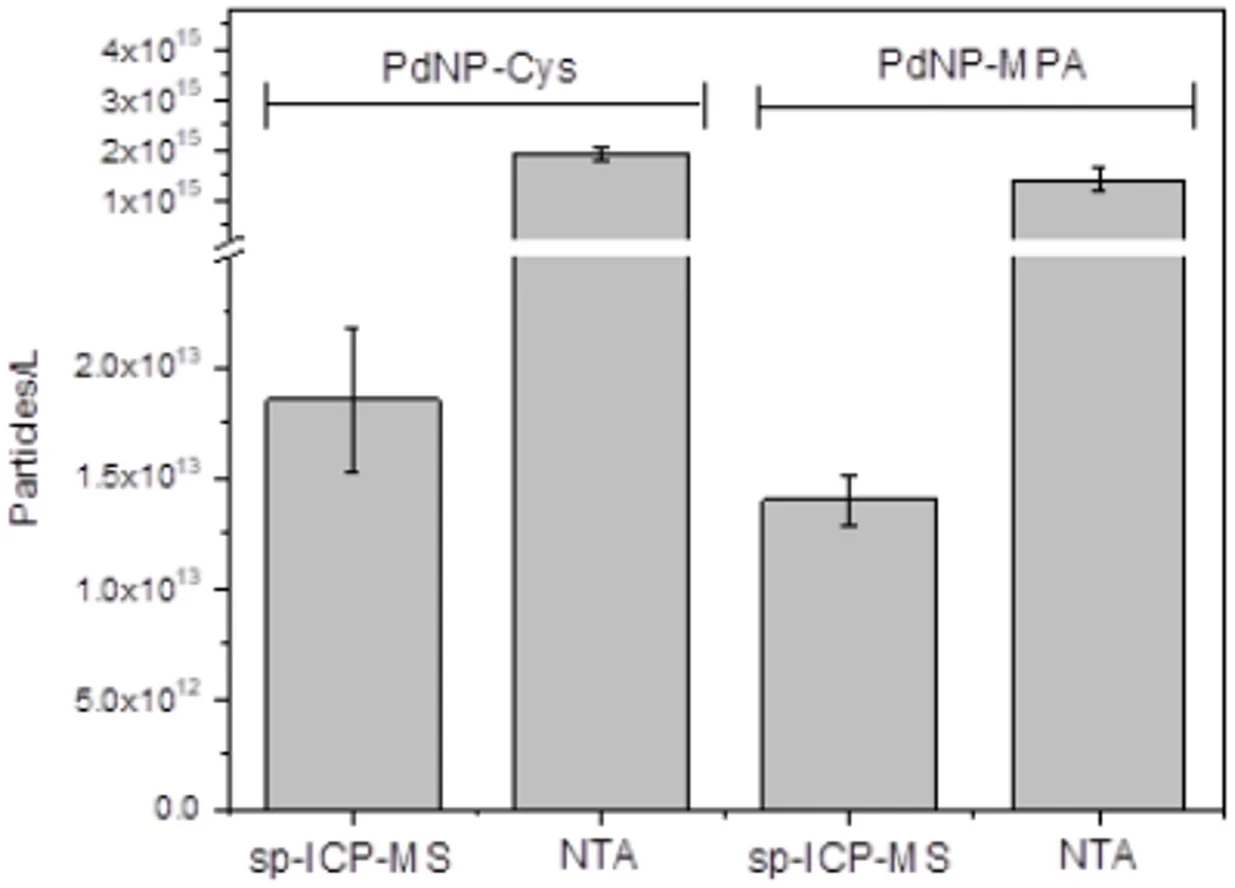
Particle number concentration in PdNP-MPA and PdNP-Cys determined by NTA and sp-ICP-MS.

The analysis of PtNP reference material was also performed by NTA (Figure S5-B). Contrary to the observed by sp-ICP-MS, the size (76 ± 33 nm) and PNC (5.6 × 10^14^ ± 1.7 × 10^13^ particles/L) obtained by NTA showed a substantial difference compared to the reference values. The observed difference most probably occurs due to the fundamental detection bias of NTA towards larger NPs or agglomerates^37^. Thus, the higher PNC measured by NTA may be due to the inclusion of non-target or particles that do not contribute to the element-specific signal in sp-ICP-MS. However, PNC determined in PdNP-MPA and PdNP-Cys by NTA and sp-ICP-MS (Fig. 5). The PNC obtained by NTA and sp-ICP-MS techniques are statistically equivalent, giving the paired t-test, at a 95% confidence level (p-value > 0.05) (Table S5).

Studies have compared the results obtained by NTA with those using sp-ICP-MS and observed that NTA may have overestimated the size and total particle number. Hellmann et al., 2024 performed the characterization of NPs reference materials (AuNPs, SiO₂, and Fe₃O₄) in ethanolic suspension using sp-ICP-MS and compared the results with those obtained by NTA^38^. According to the authors, the size distribution determined by NTA was, in general, larger than that obtained using sp-ICP-MS and showed a tendency to overestimation for small particles (< 51 nm). In general, the sizes for SiO₂, Fe₃O₄ NPs showed a good agreement with those obtained by NTA. However, for SiO₂, Fe₃O₄ NPs, the PNC determined by sp-ICP-MS were slightly closer to the certified values. This relies on the fact that, for heterogeneous NPs, NTA may not focus on all the NPs equally, leading to an overestimation of the PNC^38^.

### Ligand density assessment

The thiol ligand density of PdNP-MPA and PdNP-Cys were calculated considering a monolayer of the thiol-based ligands MPA and Cys covalently bound to NPs assumed as being of spheric shape using the mean particle sizes and PNC obtained by sp-ICP-MS and the total concentration of S and Pd measured by ICP-MS. Also, considering that nanoparticles were subjected to multiple washing cycles to remove unbound ligand, the S concentration in PdNPs was attributed to surface-bound ligands. Thereby, different approaches were used: (A) a method proposed by Elzey et al.^31^, which is based on the sulfur-to-metal ratio for determining ligand density in metallic nanoparticles, and (B) based on the stoichiometric relation of ligand and S concentration, and PNC. In the PdNPs suspensions, the total concentration of Pd was 343.80 ± 13.25 mg L^− 1^ (PdNP-MPA) and 310.33 ± 5.91 mg L^− 1^ (PdNP-Cys), respectively; the concentration of S was 0.39 ± 0.09 mg L^− 1^ (0.11 ± 0.03% (*w/w*), and 0.35 ± 0.08 mg L^− 1^ (0.11 ± 0.02% (*w/w*)), respectively, for PdNP-MPA and PdNP-Cys. These results were obtained *via* bulk ICP-MS analysis and are in good agreement with those obtained *via* TEM/EDS (PdNP-MPA (0.21 ± 0.11% (*w/w*)) and PdNP-Cys (0.14 ± 0.11% (*w/w*)), as mentioned above in section *Structure and composition.*

**Fig. 6:**
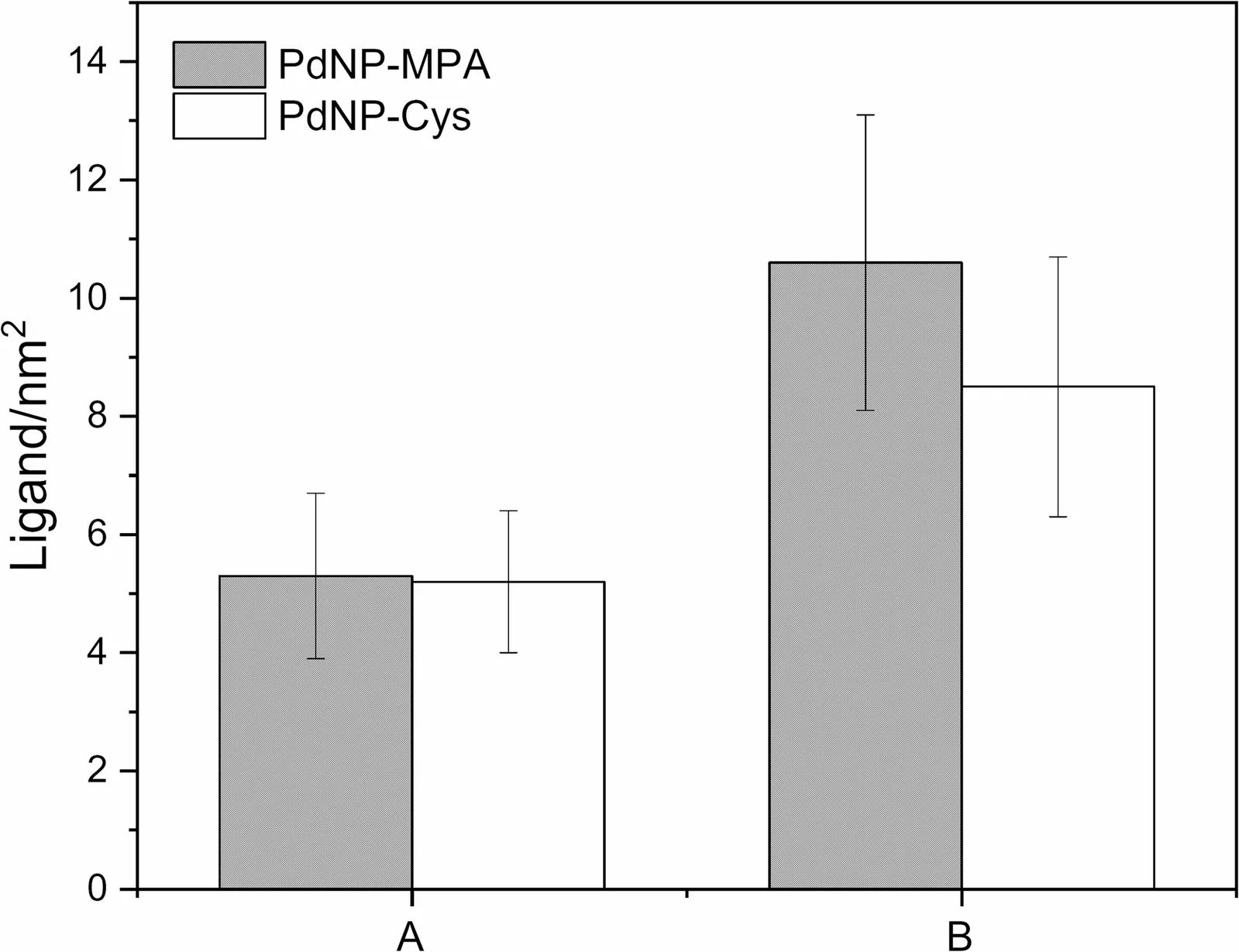
Ligand density assuming spherical shaped PdNPs and calculation by (A) S/Pd ratio and (B) the stoichiometric relation of ligand, S concentration and total particle number.

The ligand density obtained considering the two stated approaches and assuming spherical shaped particles are shown in Fig. 6. The ligand density based on the S/Pd ratio was slightly lower than the ligand density obtained by the ligand stoichiometric relation and PNC. However, according to ANOVA, at a 95% confidence level (Table S6), there is no significant difference (p-value > 0.05) among the values of ligand density obtained by the different calculation approaches. Using the S/Pd ratio for ligand density estimation is particularly interesting because the number of particles does not affect the calculation, and any potential NPs loss during ligand removal may have no influence on the results^31^.

However, it is important to highlight that particle morphology and polydispersity have an important influence on the calculation of the ligand density. Considering spherical, cubic, isooctahedral, and dodecahedral shaped particles and assuming particles with the same volume, the spherical ones have the lowest surface-to-volume ratio and tend to show the highest average ligand density. Consequently, particles with the same volume, the non-spheric PdNP-MPA and PdNP-Cys will probably show a slightly lower ligand density than those observed for spherical particles.

### Conclusion and outlook

**Fig. 7:**
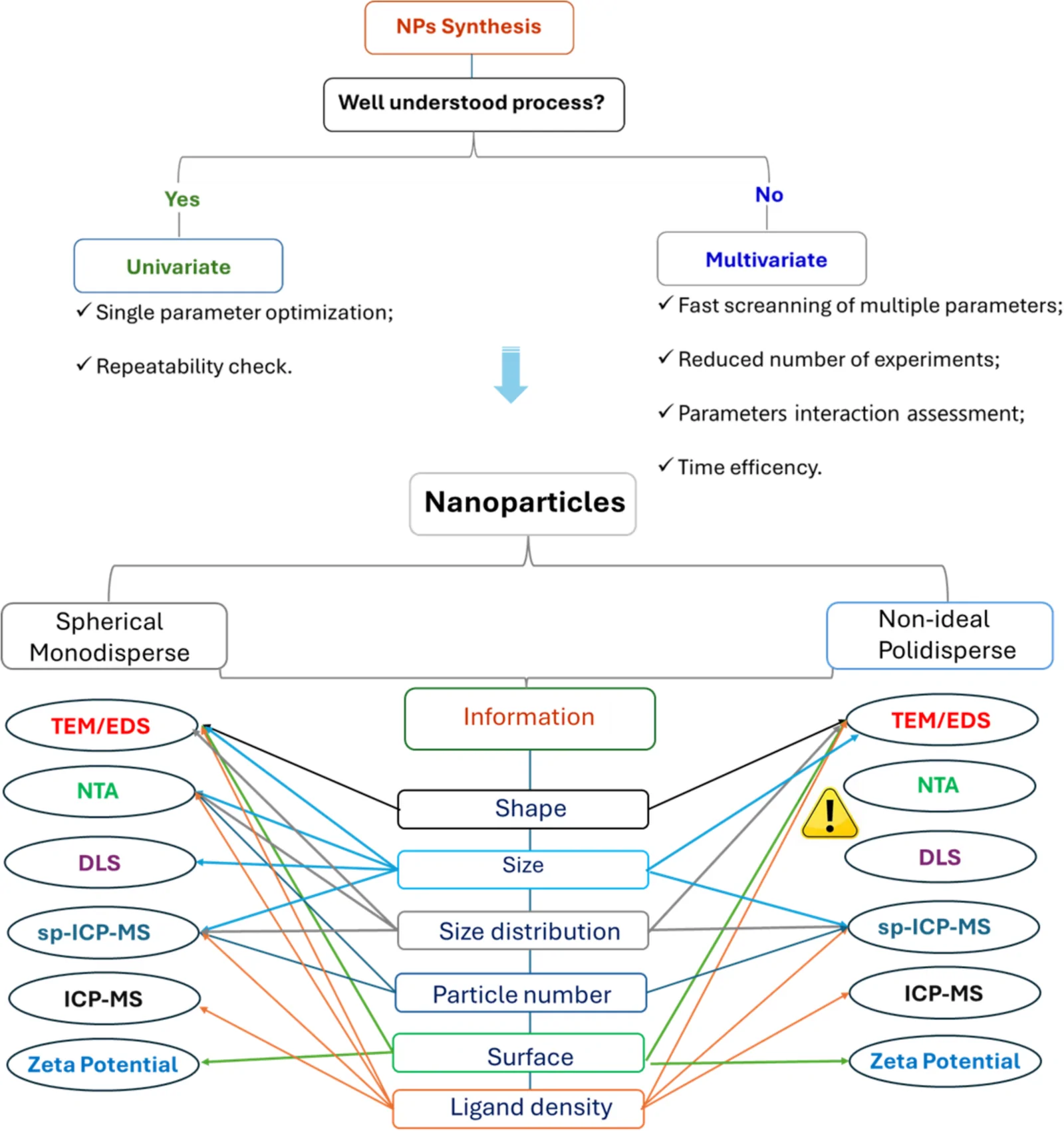
Decision-tree framework for the optimization of the NPs synthesis and characterization using a multi-technique approach. Yellow warning symbol indicates critical decision point associated with incomplete or biased results for non-ideal NPs using solely NTA or DLS analysis.

Overall, our study demonstrated the (i) potential of multivariate approaches to identify and optimize the factors influencing NP formation, throughout the synthesis of PdNP-MPA and PdNP-Cys NPs taking sustainability into account, (ii) advantages of a multi-analytical approach to a comprehensive characterization of NPs, providing results of high quality and good reproducibility, (iii) and the applicability of ICP-MS based techniques for NPs comprehensive size-, surface-characterization and number-based quantification. The PdNP-MPA and PdNP-Cys, used as proof-of-concept showed to be interesting model systems for real world NP samples encountered, e.g., in environmental analysis, which reveal a broad size and shape distribution. Thus, to provide conceptual assistance and promote coherent selection of uni/multivariate approaches for NPs synthesis optimization, and complementary multi-technique characterization strategies for non-ideal NP systems, a decision-tree framework is suggested below (Fig. 7).

The establishment of NPs synthesis conditions is an important step that will drive the structural and compositional characteristics of the particles. For well-known synthesis the univariate approach would be a useful, fast, and efficient way for particle synthesis. However, if the synthesis process is not fully understood or for tailored NPs, a multivariate approach for NPs synthesis optimization allows the simultaneous evaluation of the most significant parameters in a time efficient way. For characterization of ideal systems containing spherical and monodisperse systems, the presented techniques will be perfectly well-suited. However, for non-ideal systems one should be aware of some potential bias due to size and shape variation. The DLS and NTA analysis demonstrated satisfactory performance for the estimation of hydrodynamic diameter of PdNP-MPA and PdNP-Cys NPs. Nevertheless, in polydisperse systems DLS measurements may be biased towards larger particles. sp-ICP-MS is a powerful tool for the characterization of polydisperse and non-defined shape particles allowing for the determination of the size and size distribution; results are comparable with those obtained by complementary analytical techniques. Besides physical characterization bulk ICP-MS demonstrated to be useful for surface characterization through the ligand density determination of thiol-capped nanoparticles.

In summary, we present a NPs synthesis route in aqueous medium as well as a subsequent complementary multi-technique analytical approach for full NPs characterization. As an authors´ prediction – we believe in future work, both “sides” will closely link together: automated NPs synthesis comprising a closed feedback loop underpinned with complementary multi-technique analysis.

## Experimental section

### Reagents and solutions

All reagents used were of analytical grade and were used without further purification. Double-distilled nitric acid (HNO_3_) (65% (wt.%), Merck, Darmstadt, Germany) and hydrochloric acid (HCl) (37% (wt.%), Merck) were used for standards acidification and decomposition of NPs. For synthesis of NPs K_2_PdCl_4_ (99.99%, Sigma-Aldrich, St. Louis, EUA), L-ascorbic acid (> 99.5%, Sigma-Aldrich), L-cysteine (Cys, > 98% Sigma-Aldrich), 3-mercaptopropionic acid (MPA, 98%, Sigma-Aldrich) were used. Standard solutions of Pd 1,000 mg L^− 1^ in HNO_3_, 2–3% (Sigma-Aldrich), Pt 1,000 mg L^− 1^ (Merck), S 1,000 mg L^− 1^ (Merck), and Rh 1,000 mg L^− 1^ (Merck), as internal standard, were used.

### Synthesis of Palladium Nanoparticles (PdNPs) and surface modification

The synthesis of the PdNPs was performed following the procedure described by He et al. 2009^39^ and Lim et al. 2009^32^ with some modifications using ascorbic acid as the reducing agent and MPA and Cys as surface-stabilizing ligands. To speed up PdNP synthesis a multi-experiment device Radleys Core+ (Radleys, Saffron Walden, UK) with a stirring block (Starfish from Heidolph Schwabach, Germany) was employed that enables the simultaneous synthesis of up to 40 batches from different reaction mixtures at an identical agitation speed and temperature. Optimization of the synthesis´ parameters was done applying a complete 2^5−1^ factorial design to evaluate the significance of the following parameters: Pd (2–16 µmol), ligand (MPA) (0.02–0.14 mmol) and reducing agent (0.05–0.25 mmol) concentrations, reaction time (30–180 min), and reaction temperature (25–75 °C). The levels of the evaluated parameters and the respective coded values required for the multivariate approach are shown in Table S7 of the SI. Considering the fast reduction of Pd promoted by L-ascorbic acid^32^, to obtain a reproducible, analytically accessible and standardized synthesis procedure, the effects and interactions of the optimization parameters were evaluated *via* polydispersity index (PDI). In addition, to obtain a maximum response surface by the mathematical models employed in the multivariate optimization, for convenience and to facilitate the interpretation of the response surface the PDI values were converted to 1-PDI. (Table 1). The optimal conditions for PdNPs synthesis were then identified through Doehlert design optimization of the most significant variables (Pd concentration and reaction temperature). The code and variables for the Doehlert design optimization of PdNPs synthesis, capped with MPA and Cys, are shown in Table 2.

Under compromise conditions, the PdNPs were obtained as follows: For MPA capped PdNPs (PdNP-MPA), an aliquot of 150 µL of ascorbic acid solution 1.0 mol L^− 1^ (0.15 mmol) was diluted in 2.05 mL of ultrapure water, heated at 60 ± 3 °C, under 700 rpm stirring, addition of 156 µL of K_2_PdCl_4_ 0.064 mol L^− 1^ (10 µmol) and kept reacting for 10 min. Subsequently, an aliquot of 143 µL of MPA 0.56 mol L^− 1^ (0.08 mmol) was added, and the system was maintained under stirring at 60 ± 3 °C for 80 min. For PdNP capped with Cys (PdNP-Cys), the procedure was the same as described above. However, the dilution of ascorbic acid (0.15 mmol) was performed in 2.03 mL of ultrapure water, and an aliquot of 161 µL of Cys 0.50 mol L^− 1^ (0.08 mmol) was added as ligand.

### Purification of PdNPs

The obtained PdNP-MPA and PdNP-Cys were purified to remove unbound ligands through six cleaning cycles. The colloidal suspension of the synthetized PdNPs (2.5 mL) were sonicated for 2 min in an ultrasonic bath (Allpax, PTIC-5-LA, 120 W, 50 Hz), transferred to an Amicon ultrafiltration cell using a regenerated cellulose membrane with a cut-off of 10 kDa (Merck) and centrifuged at 7,000 rcf for 10 min (Eppendorf model 5810R), the supernatant solution was removed. Next, 2.5 mL of ultrapure water was added to the sample and centrifuged (7,000 rcf, 10 min) once more. For efficient unbound ligands, this step was repeated 6-times. Then, the cleaned PdNP-MPA and PdNP-Cys were redispersed in 2.5 mL of ultrapure water, S concentration determined in particles was attributed to surface-bounded ligands. To increase the recovery of PdNPs from the filtration membrane, the samples were sonicated for 1 min before being transferred to the Amber storage flasks. The schematic representation of the cleaning step procedure to remove the unbounded ligand is shown in the supplementary information (Figure S7).

### Transmission electron microscopy (TEM)

The characterization of the PdNP-MPA and PdNP-Cys was performed using a microscope JEOL JEM-2100 F-UHR, with a field emission gun and operated at 200 kV, equipped with energy-dispersive spectroscopy (EDS) system. One drop (10 µL) of the PdNPs suspension, previously sonicated for 2 min and 3-times diluted, were cast onto carbon-coated copper grids (Merck, 200 mesh) and air-dried overnight under ambient conditions. The imaging processing was performed with ImageJ (Version: 1.54 g, https://imagej.org) and the mean particle size and the size distribution were obtained considering at least 200 particles.

### Dynamic light scattering and zeta potential measurements

The dynamic light scattering (DLS) and zeta potential measurements of the PdNP-MPA and PdNP-Cys were performed at 25 °C using a Zetasizer Nano ZS (Malvern Panalytical Ltd) back scattering angle 173°, equipped with a 633 nm laser *via* disposable cells. The measurements were performed in triplicate, the z-average and number distribution were considered to determine the hydrodynamic diameter. The particle electrophoretic mobility using the Einstein–Smoluchowski theory, with a refractive index of 1.33, were used to determine the zeta potential.

### Nanoparticle tracking analysis

Nanoparticle tracking analyses (NTA) were carried out using a NanoSight LM10 (Malvern Panalytical, UK) equipped with a sCMOS camera and a blue 405 nm laser to determine the number-based hydrodynamic diameter (dh,_0_) and the particle number concentrations (PNC). All measurements were performed at a temperature of 25 °C in static mode and data analysis was performed with the NanoSight NTA software (Version: 3.32), that captured 5 videos with 60 s and 25 fps of laser light scattering of the highly diluted samples, as well as manual shutter and gain adjustments.

### Bulk ICP-MS and single particle-ICP-MS analysis

The total concentration of S and Pd in PdNP-MPA and PdNP-Cys was determined *via* an ICP-MS Agilent 8900 (ICP-QQQ, Agilent Technologies, Japan) equipped with a quartz spray chamber and MicroMist nebulizer, after sample decomposition by means of *aqua regia*. In brief, an aliquot of 1 mL of the colloidal suspension was centrifuged for 10 min at 13,000 rpm (Sigma, model 1–14 K), 950 µL of the supernatant was removed and 200 µL of freshly prepared *aqua regia* was added to the sedimented NPs. The mixture was sonicated for 15 min at room temperature and, after NPs decomposition, 750 µL of ultra-pure water was added. The samples were diluted 250 and 20,000-times for determination of S and Pd respectively. The calibration was performed with aqueous standards from 0.1 to 50 µg L^− 1^ using Rh (2 µg L^− 1^) as internal standard. The measurements were performed in triple quadrupole mode with Q1 and Q3 set to *m/z* 105 for Pd, for S these parameters were set to *m/z* 32 (Q1) and *m/z* 48 (Q3), using a dwell time of 100 ms. The CRC was pressurizing with He (5.5 mL min^− 1^) and O_2_ (0.3 mL min^− 1^) for measuring Pd, and S respectively.

sp-ICP-MS measurements were performed with a dwell time of 100 µs with a total acquisition time of 180 s. Transport efficiency (TE) was obtained by measuring a reference of platinum nanoparticles (PtNPs) of known particle size (50 nm) as well as ionic standards of Pt at 1 µg L^− 1^ and blank solutions, both containing 1% (*v/v*) HCl (37% (wt.%)), and 0.5% (*v/v*) HNO_3_ (65% (wt.%)). Prior to the measurements, the reference PdNPs and the synthesized PdNPs were sonicated for 1 min, diluted in ultrapure water.

The limits of detection for total concentration of Pd and S were determined by 10 consecutive measurements of blank solutions, divided by the slope of the respective calibration curve. The limits of detection for size and dissolved concentration for PdNPs were determined according to Laborda et al. 2020^40^. The instrumental parameters for the ICP-MS and sp-ICP-MS measurements as well as the respective limits of detection are shown in Table S8.

### Ligand density assessment

The thiol ligand density of PdNP-MPA and PdNP-Cys were estimated considering a monolayer of the thiol-based ligands MPA and Cys covalently bound to NPs assumed as being of spheric shape using the mean particle sizes and particle number concentrations obtained by sp-ICP-MS and considering the total concentration of S and Pd determined by ICP-MS. Thereby, different approaches were evaluated: (A) a method proposed by Elzey et al.^34^, which is based on the sulfur-to-metal ratio for determining ligand density in metallic nanoparticles and, (B) based on the stoichiometric relation of ligand and S concentration, and the PNC.

## Supplementary Information

Below is the link to the electronic supplementary material.


Supplementary Material 1


## Data Availability

All data generated during this study are included in this published article, in the supplementary information files or are available from the corresponding authors on reasonable request.
