# Supplementary material for: Multivariate synthesis optimization, comprehensive characterization, and surface ligand determination of palladium nanoparticles
Source: Sci Rep. 2026 Sep 2;16:27501. doi: 10.1038/s41598-026-69103-3 (PMC13534612; doi:10.1038/s41598-026-69103-3)
Supplement: Supplementary file 1 [file 41598_2026_69103_MOESM1_ESM.docx]

***Supplementary material for***

**Multivariate synthesis optimization, comprehensive characterization, and surface ligand determination assessed for thiol-capped palladium nanoparticles**

Eduardo Sidinei Chaves ^1,3*^, Morgana Lurdes da Rocha^1^, Isabella Tavernaro^2^,

Ute Resch-Genger^2*^ and Björn Meermann^3*^

^1^Federal University of Santa Catarina, Department of Chemistry, Florianópolis, Brazil.

^2^Federal Institute for Materials Research and Testing, Division 1.2- Biophotonics, Berlin, Germany.

^2^Federal Institute for Materials Research and Testing, Division 1.1- Inorganic Trace Analysis (ITALab), Berlin, Germany.

* Corresponding authors:	[eduardo.chaves@ufsc.br](mailto:eduardo.chaves@ufsc.br)

[ute.resch@bam.de](mailto:ute.resch@bam.de)

[bjoern.meermann@bam.de](mailto:bjoern.meermann@bam.de)

***Content:***

***Tables S1 to S10***

***Figures S1 to S7***

**Figure S1.** Pareto´s Chart for the fractionated factorial design 2^5-1^ optimization of the water-based PdNPs synthesis. Effect of the synthesis´ parameters of the PdNPs, at a confidence level of 95%, significant effect on the PDI showed p<0.05.

**Table S****1.** ANOVA of the 2⁵⁻¹ fractional factorial design for screening the synthesis parameters of PdNPs capped with a thiol-based ligand

| Fator | SS | df | MS | F | p |
| --- | --- | --- | --- | --- | --- |
| Curvature | 0.006760 | 1 | 0.006760 | 33.0841 | 0.028921 |
| (1) Temperature, ^o^C | 0.001139 | 1 | 0.001139 | 5.5745 | 0.142120 |
| (2) Time, min | 0.000000 | 1 | 0.000000 | 0.0003 | 0,987634 |
| (3) Pd, µ mol | 0.001425 | 1 | 0.001425 | 6.9742 | 0.118445 |
| (4) Ligand, m mol | 0.000116 | 1 | 0.000116 | 0.5656 | 0.530487 |
| (5) Ascorbic acid, m mol | 0.000410 | 1 | 0.000410 | 2.0068 | 0.292291 |
| 1 by 2 | 0.000315 | 1 | 0.000315 | 1.5419 | 0,340203 |
| 1 by 3 | 0.033398 | 1 | 0.033398 | 163.4465 | 0.006063 |
| 1 by 4 | 0,000333 | 1 | 0.000333 | 1.6300 | 0.329899 |
| 1 by 5 | 0.000798 | 1 | 0.000798 | 3.9057 | 0.186770 |
| 2 by 3 | 0.000689 | 1 | 0.000689 | 3.3722 | 0.207715 |
| 2 by 4 | 0.000770 | 1 | 0.000770 | 3.7687 | 0.191731 |
| 2 by 5 | 0.000028 | 1 | 0.000028 | 0.1349 | 0.748637 |
| 3 by 5 | 0.001661 | 1 | 0.001661 | 8.1267 | 0.104175 |
| 3 by 5 | 0.001073 | 1 | 0.001073 | 5.2491 | 0.149058 |
| 4 by 5 | 0.000086 | 1 | 0.000086 | 0.4187 | 0.583919 |
| Pure error | 0.000409 | 2 | 0.000204 |  |  |
| Total SS | 0.006760 | 18 |  |  |  |

R^2^:0.99173; Adj.: 0.92556; MS Pure error:0.0002043.

Ligand: 3-mercaptopropionic acid

**Table S2.** ANOVA of the Doehlert design of the PdNPs synthesis capped with 3-mercaptopropionic acid (MPA) based on the 1-PDI.

| Fator | SS | df | MS | F | p |
| --- | --- | --- | --- | --- | --- |
| (1) Pd, µ mol (L) | 0.001752 | 1 | 0.001752 | 2.666794 | 0.244063 |
| Pd, µ mol (Q) | 0.000389 | 1 | 0.000389 | 0.591781 | 0.522161 |
| (2) Temperature, ^o^C(L) | 0.001482 | 1 | 0.001482 | 2.256088 | 0.271931 |
| Temperature, ^o^C(Q) | 0.000128 | 1 | 0.000128 | 0.195028 | 0.701923 |
| 1L by 2L | 0.000020 | 1 | 0.000020 | 0.030822 | 0.876805 |
| Lack of fit | 0.000840 | 1 | 0.000840 | 1.278792 | 0.375485 |
| Pure error | 0.001314 | 2 | 0.000657 |  |  |
| Total SS | 0.006040 | 8 |  |  |  |

R^2^:0.64366; Adj.: 0.04897; 2 factors;1 block, 9 runs; MS Pure error:0.000657.

**Table S3.** ANOVA of the Doehlert design of the PdNPs synthesis capped with L-cysteine based on the 1-PDI.

| Fator | SS | df | MS | F | p |
| --- | --- | --- | --- | --- | --- |
| (1) Pd, µ mol (L) | 0.016576 | 1 | 0.016576 | 12.10540 | 0.073604 |
| Pd, µ mol (Q) | 0.002803 | 1 | 0.002803 | 2.04722 | 0.288780 |
| (2) Temperature, ^o^C(L) | 0.000121 | 1 | 0.000121 | 0. 08836 | 0.794300 |
| Temperature, ^o^C(Q) | 0.005603 | 1 | 0.005603 | 4.09202 | 0.180426 |
| 1L by 2L | 0.000784 | 1 | 0.000784 | 0.57254 | 0.528239 |
| Lack of fit | 0.000451 | 1 | 0.000451 | 0.32911 | 0.624095 |
| Pure error | 0.002739 | 2 | 0.001369 |  |  |
| Total SS | 0.027776 | 8 |  |  |  |

R^2^:0.88518; Adj.: 0.69381; 2 factors;1 block, 9 runs; MS Pure error:0.0013693.

**Figure S2.** Sulfur concentration in the PdNP-MPA and PdNP-Cys supernatant according to the washing cycles.

**Table S4.** ANOVA from pared t-test with 95% of confidence, comparing NTA and DLS data

|  | *DLS* | *NTA* |
| --- | --- | --- |
| Mean | 136 | 127,5 |
| Variance | 1250 | 480,5 |
| Observations | 2 | 2 |
| Pearson Correlation | 1 |  |
| Hypothesized Mean Difference | 0 |  |
| df | 1 |  |
| T Stat t | 0.894737 |  |
| P(T<=t) one-tail | 0.267666 |  |
| t critical one-tail | 6.313752 |  |
| P(T<=t) two-tail | 0.535331 |  |
| t critical two-tail | 12.7062 |  |

**Figure S3.** DLS number-based size distribution for PdNP-MPA and PdNP-Cys.

**Figure S4.** NTA particle size distribution for PdNP-MPA and PdNP-Cys.

**Figure S5.** Size, size distribution and particle number obtained by (A) sp-ICP-MS and (B) NTA for reference material of PtNP.

**Figure S6.** Dilution effect in the (A) particle size and (B) number obtained by sp-ICP-MS.

**Table S5.** ANOVA from pared t-test with 95% of confidence, comparing NTA and sp-ICP-MS data

|  | *Sp-ICP-MS* | *NTA* |
| --- | --- | --- |
| Mean | 1.63E+13 | 1.68E+15 |
| Variance | 1.01E+25 | 1.35E+29 |
| Observations | 2 | 2 |
| Pearson Correlation | 1 |  |
| Hypothesized Mean Difference | 0 |  |
| df | 1 |  |
| T Stat t | -6.4549 |  |
| P(T<=t) one-tail | 0.048924 |  |
| t critical one-tail | 6.313752 |  |
| P(T<=t) two-tail | 0.097848 |  |
| t critical two-tail | 12.7062 |  |

**Table S6.** ANOVA one-way with 95% of confidence, comparing the ligand density considering PdNP with different morphologies.

| *Source of Variation* | *SS* | *Df* | *MS* | *F* | *P-value* | *F crit* |
| --- | --- | --- | --- | --- | --- | --- |
| Between Groups | 18.30525742 | 4 | 4.576314354 | 1.609253 | 0.246545 | 3.47805 |
| Within Groups | 28.43750867 | 10 | 2.843750867 |  |  |  |
| Total | 46.74276609 | 14 |  |  |  |  |

**Table S7**. Variables, coded values, and levels for PdNPs synthesis by 2^5-1^ fractionated factorial design.

| **Variables** | **Levels** | | |
| --- | --- | --- | --- |
|  | **-1** | **0** | **+1** |
| Temperature, ^o^C | 25 | 50 | 75 |
| Time, min. | 30 | 105 | 180 |
| Pd, µmol | 2 | 9 | 16 |
| Ligand, mmol | 0.02 | 0.08 | 0.14 |
| Ascorbic acid, mmol | 0.05 | 0.15 | 0.25 |

**Figure S7.** Schematic representation of the unbounded ligand removal of the PdNP-MPA and PdNP-Cys

**Table S8.** Instrumental parameters for the ICP-MS and sp-ICP-MS measurements.

| RF power, W | 1550 | |
| --- | --- | --- |
| Plasma gas flow rate, L min^-1^ | 15.0 | |
| Auxiliary gas flow rate L min^-1^ | 0.90 | |
| Nebulizer | Concentric | |
| sample uptake, mL min^-1^ | 0.346 | |
|  | ***sp-ICP-MS mode*** | ***ICP-MS mode*** |
| Torch injector | 1.5 mm | 2.5 mm |
| Dwell time, ms | 0.1 | 100 |
| Collision / Reaction gas | No gas | He: 5.5 mL min^-1^(Pd)  O_2_: 0.3 mL min^-1^ (S) |
| Gas flow nebulizer, L min^-1^ | 0.80 | 1.07 |
| Monitored isotopes | ^105^Pd, ^194^Pt | ^32^S, ³²S¹⁶O⁺, ^105^Pd, ^103^Rh (IS) |
|  |  |  |
| Limit of detection | LOD_size_ 30 nm | Pd 10 ng L^-1^ |
|  | LOD_conc._ 1.3 ng L^-1^ | Pd 17 ng L^-1^ |
|  |  |  |
